# The effects of artocarpin on wound healing: *in vitro* and *in vivo* studies

**DOI:** 10.1038/s41598-017-15876-7

**Published:** 2017-11-15

**Authors:** Chung-Ju Yeh, Chin-Chuan Chen, Yann-Lii Leu, Ming-Wei Lin, Mei-Miao Chiu, Shu-Huei Wang

**Affiliations:** 1 Graduate Institute of Natural Products, Chang Gung University, Taoyuan, Taiwan; 2 Chinese Herbal Medicine Research Team, Healthy Aging Research Center, Chang Gung University, Taoyuan, Taiwan; 3 Tissue Bank, Chang Gung Memorial Hospital, Taoyuan, Taiwan; 4 Department of Laboratory Medicine, National Taiwan University Hospital, Taipei, Taiwan; 5 Department of Medicine, Mackay Medical College, New Taipei, Taiwan, Republic of China; 6 Department of Anatomy and Cell Biology, College of Medicine, National Taiwan University, Taipei, Taiwan

## Abstract

The skin protects the body against harmful substances and microorganisms. When the skin is damaged, wound healing must be finely regulated to restore the normal function of skin tissue. Artocarpin (ARTO), a prenylated flavonoid purified from the plant *Artocarpus communis*, has been reported to have anti-inflammatory and anti-cancer properties. The aim of the present study was to evaluate the wound healing potential and therapeutic mechanism of ARTO. Immunohistochemical staining of neutrophils and macrophages and mouse cytokine array analysis demonstrated that ARTO accelerates inflammatory progression and subsequently decreases persistent inflammation. ARTO increases collagen production and increases human fibroblast proliferation and migration by activating the P38 and JNK pathways. Moreover, ARTO increases the proliferation and migration of human keratinocytes through the ERK and P38 pathways and augments human endothelial cell proliferation and tube formation through the Akt and P38 pathways. Together, our data suggested that ARTO enhances skin wound healing, possibly by accelerating the inflammatory phase and by increasing myofibroblast differentiation, proliferation and migration of fibroblasts and keratinocytes, collagen synthesis and maturation, re-epithelialization, and angiogenesis. These findings indicate that ARTO has potential as a potent therapeutic agent for the treatment of skin wounds.

## Introduction

The skin serves as the body’s first line of defense and protects the body from various harmful agents, such as chemicals, heat, UV radiation, and microorganisms. When the skin is damaged, its protective function is lost, and the body must generate new tissue to restore this function within a short period of time. In most cases, this regeneration is accomplished through a well-regulated wound healing process. However, if the wound is not rapidly healed, microbial infection may occur. In more severe cases, amputation may even be necessary. Therefore, determining how to accelerate the wound healing process is an important issue.

Wound healing is a dynamic and complex process characterized by three main overlapping phases: the inflammatory phase, the proliferative phase, and the remodeling phase. In the inflammatory phase, neutrophils and macrophages are attracted to the wound area, where they phagocytose foreign particles, microbes, and damaged tissue^1,2^. In the proliferative phase, fibroblasts proliferate and migrate to the wound area and secrete new extracellular matrix (ECM), forming granulation tissue^2,3^. Re-epithelialization also occurs in this phase. In this process, keratinocytes proliferate and migrate from the wound edges to the center of the wound^4,5^. Angiogenesis is an important part of the proliferative phase of the wound healing process because it establishes blood supply to the newly formed tissue^4^. Finally, in the remodeling phase, the ECM matures. In particular, collagen bundles increase in diameter and become more organized, thereby providing more tensile strength^4,6^. Fibroblasts also transform into myofibroblasts and thus facilitate wound contraction^6–8^. Excess ECM is subsequently removed, and the wound healing process is completed^1,3^.

**Figure 1:**
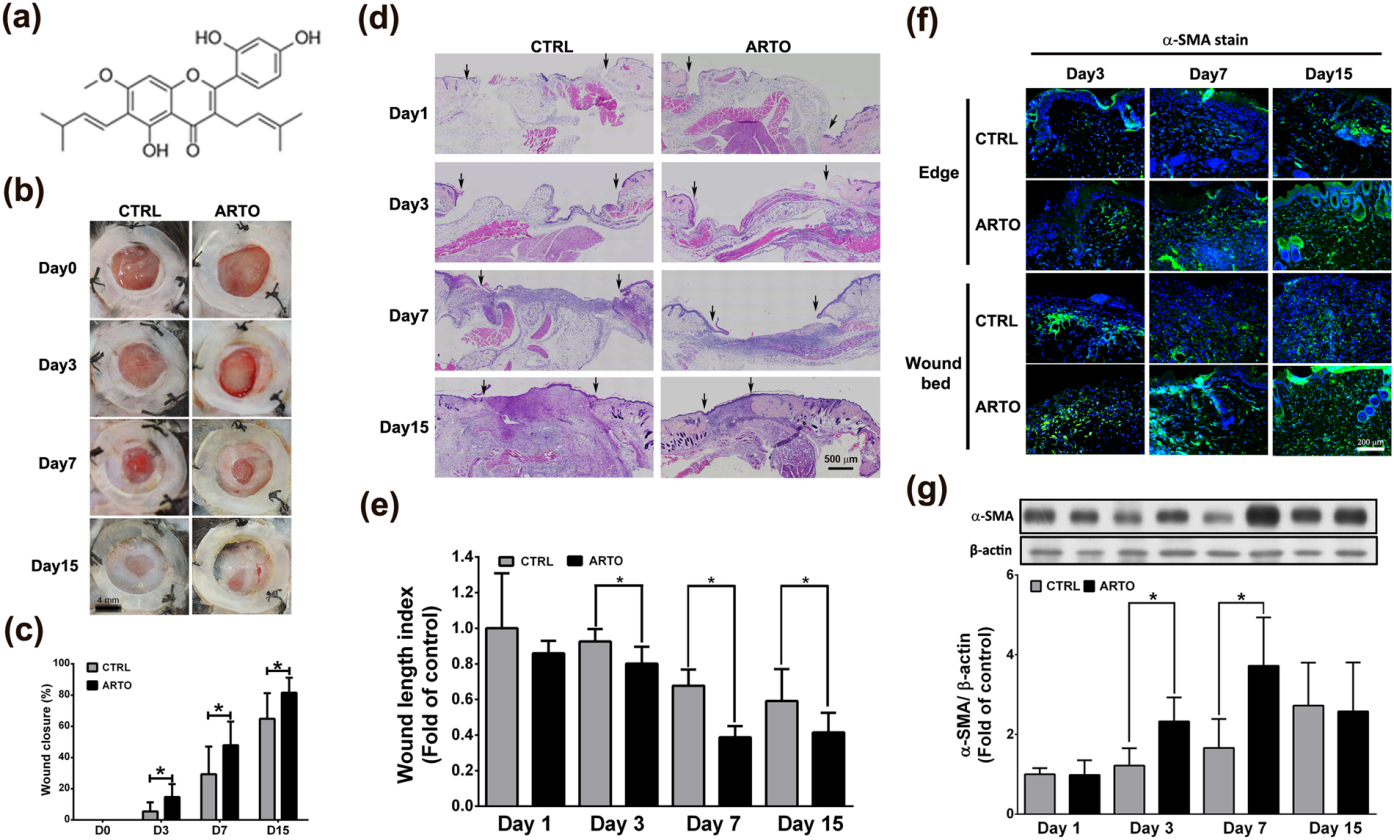
ARTO accelerates skin contraction and wound healing. (**a**) The structure of ARTO. (**b**,**c**) Representative photographs of wound healing in CTRL and ARTO-treated mice at various time points after wounding. The wound closure results were quantified on days 3, 7, and 15 after wounding. (**d**,**e**) H&E-stained sections on days 1, 3, 7, and 15 after wounding. The widths of the wound areas are marked by arrows and were quantified on days 1, 3, 7, and 15 after wounding. (**f**) Immunohistochemistry was performed to identify α-SMA in wounds on days 3, 7, and 15 after wounding. (**g**) The α-SMA levels were determined by western blot analysis. The data are shown as the means ± S.D. N = 6–18 wounds/group and *P < 0.05.

Artocarpin (ARTO) (Fig. 1a) is a prenylated flavonoid purified from the plant *Artocarpus communis* ^9^. Previous studies have shown that ARTO has anti-inflammatory^9–11^, anti-oxidative^9^, anti-cancer^12,13^, and anti-microbial^14,15^ properties. However, ARTO’s effects on skin wound healing remain unknown and have not been reported to date. In the present study, we observed that ARTO accelerates the wound healing process by accelerating the inflammatory phase progression and by enhancing collagen deposition, re-epithelialization, and angiogenesis. Our data indicated that ARTO has potential as a therapeutic agent for the treatment of cutaneous wounds.

## Results

### ARTO accelerates skin wound healing

To investigate the effects of ARTO on skin wound healing, we first used an excisional wound model to observe the healing process in C57BL/6 mice. The wound closure percentage was significantly higher in the ARTO-treated group than in the control (vehicle control) group on days 3, 7, and 15 after wounding (Fig. 1b,c).

Wound contraction^4,6^ and myofibroblasts^8^ both play important roles during the remodeling phase of wound healing. In hematoxylin and eosin (H&E)-stained sections, we observed shorter distances between the wound edges in the ARTO-treated group on days 3, 7, and 15 (Fig. 1d,e). Immunohistochemistry and western blot analysis also showed that the level of alpha smooth muscle actin (α-SMA), a myofibroblast-specific marker, was significantly higher in the ARTO-treated group on days 3 and 7 after wounding. However, the increasing effect was decreased on day 15 after wounding (Fig. 1f,g). Together, these results indicated that ARTO accelerates skin wound healing by enhancing myofibroblast level and increasing wound contraction during the remodeling phase of the wound healing process.

### ARTO accelerates inflammatory phase progression and decreases the inflammatory responses of wound healing

**Figure 2:**
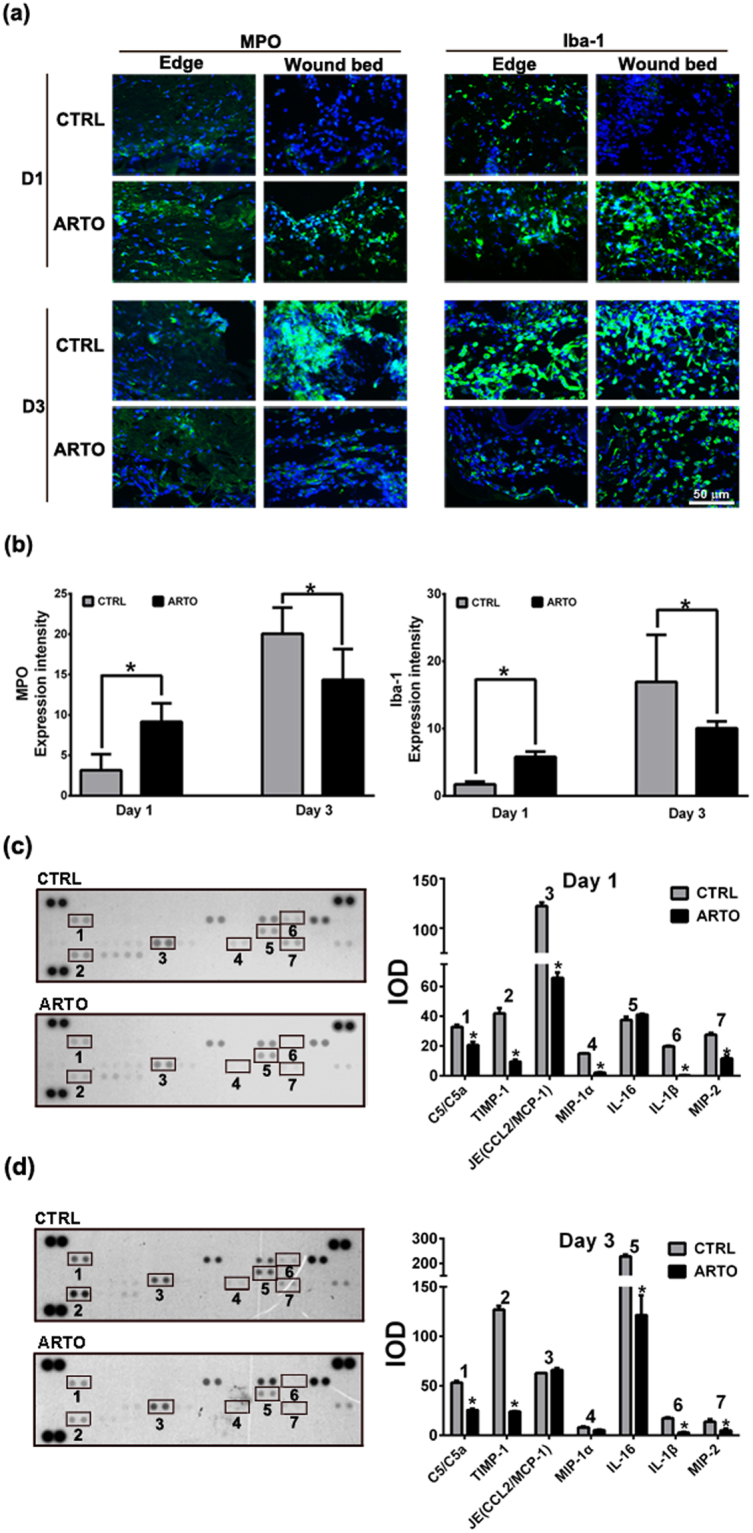
ARTO accelerates inflammatory phase progression and decreases the inflammatory responses of wound healing. (**a**) Immunohistochemistry was performed to identify infiltrating neutrophils (MPO) and macrophages (Iba-1) in wounds on days 1 and 3. (**b**,**c**) Quantitative MPO and Iba-1 level on days 1 and 3 after wounding. (**c**,**d**) Protein extracts of the skin wounds were collected on day 1 and day 3 for use in cytokine membrane array assays (left panel). The cytokine level was quantified (right panel). The data are shown as the means ± S.D. N = 3–6 wounds/group and *P < 0.05.

The recruitment of inflammatory cells, such as neutrophils and macrophages, to the wound area is an important event in the inflammatory phase of wound healing. In the present study, immunohistochemistry showed higher numbers of neutrophils and macrophages in the wound area in the ARTO-treated group than the control group on day 1 after injury (Fig. 2a,b). However, on day 3, there were fewer neutrophils and macrophages in the ARTO-treated group than in the control group. Additionally, mouse cytokine arrays showed that the levels of C5/C5a, tissue inhibitor of metalloproteinases 1 (TIMP-1), monocyte chemotactic protein 1 (MCP-1), macrophage inflammatory protein 1 alpha, 2 (MIP-1α, 2), interleukin 16 (IL-16), and interleukin 1 beta (IL-1β) were decreased in the ARTO-treated group on day 1 or day 3 after injury (Fig. 2c,d). Together, these data suggested that ARTO accelerates inflammatory phase progression by causing an early peak of inflammation with accelerated infiltration and elimination of inflammatory cells and later decreases persistent inflammation.

### ARTO enhances collagen maturation and deposition

**Figure 3:**
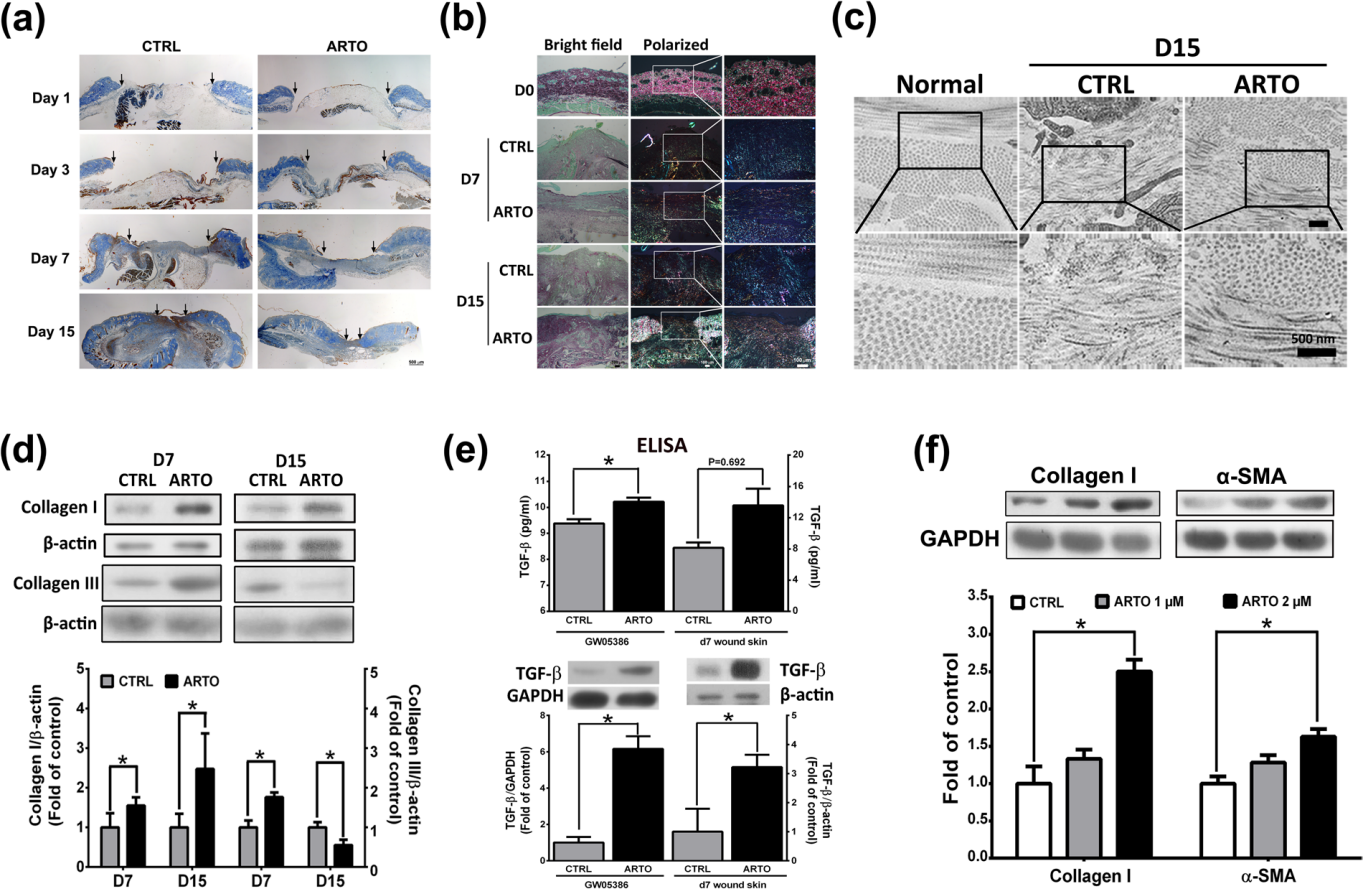
ARTO enhances collagen deposition and maturation. The wounded sections stained with Mallory’s trichrome (**a**) and picrosirius red (**b**) showed that more newly formed and mature collagen fibers were observed in ARTO-treated wounds. The widths of the wound areas are marked by arrows. (**c**) TEM images of connective tissue on day 15 after wounding. Higher magnification images (lower panel) illustrate different collagen fibril diameters, amounts, and arrangements in skin wounds. (**d**) Western blot analysis of the skin showed collagen deposition (collagen type I and III) on days 7 and 15 after wounding. (**e**) TGF-β level was analyzed in GM05386 fibroblasts and skin by ELISA and western blot. (**f**) The collagen I and α-SMA levels were determined by western blot analysis. The data are shown as the means ± S.D. N = 3–6 wounds/group and *P < 0.05.

The second phase of wound healing is the proliferative phase, in which collagen deposition plays an important role^4^. In the current study, Mallory’s aniline blue stain (Fig. 3a) indicated an increase in the amount of collagen in the ARTO-treated group. Picrosirius red staining was performed to observe collagen maturation. In picrosirius red staining, mature collagen (collagen I) appears yellow to red under a polarizing microscope, and immature collagen (collagen III) appears green. We observed more abundant and mature collagen in the ARTO-treated group than in the control group on days 7 and 15 (Fig. 3b). Transmission electron microscopy (TEM) also showed thicker collagen deposition and more regular and uniform collagen arrangement in the ARTO-treated group than in the control group on day 15 (Fig. 3c). In addition, western blot analysis also showed that the collagen I level was significantly higher in the ARTO-treated group on days 7 and 15. The collagen III level was significantly higher in the ARTO-treated group on day 7 but was lower in the ARTO-treated group on day 15 (Fig. 3d).

Fibroblasts are responsible for new ECM formation and collagen deposition in the wound area^16,17^. Previous studies have shown that transforming growth factor beta (TGF-β) enhances fibroblast proliferation and collagen deposition^18^. In the present study, ELISA and western blot analysis showed that TGF-β level was increased in ARTO-treated GM05386 fibroblasts (human fibroblasts) and in ARTO-treated wounded skin on day 7 after injury (Fig. 3e). To determine the effects of ARTO on collagen synthesis and myofibroblast differentiation in fibroblasts, GM05386 fibroblasts were subsequently used for experimentation, and western blotting showed a significantly higher collagen type I and α-SMA level in the ARTO-treated group than the control group (Fig. 3f). Together, these results indicated that ARTO accelerates wound healing by enhancing collagen deposition and myofibroblast differentiation and that this effect may be due to increased TGF-β level.

### ARTO enhances human fibroblast proliferation through the P38 and JNK signaling pathways

**Figure 4:**
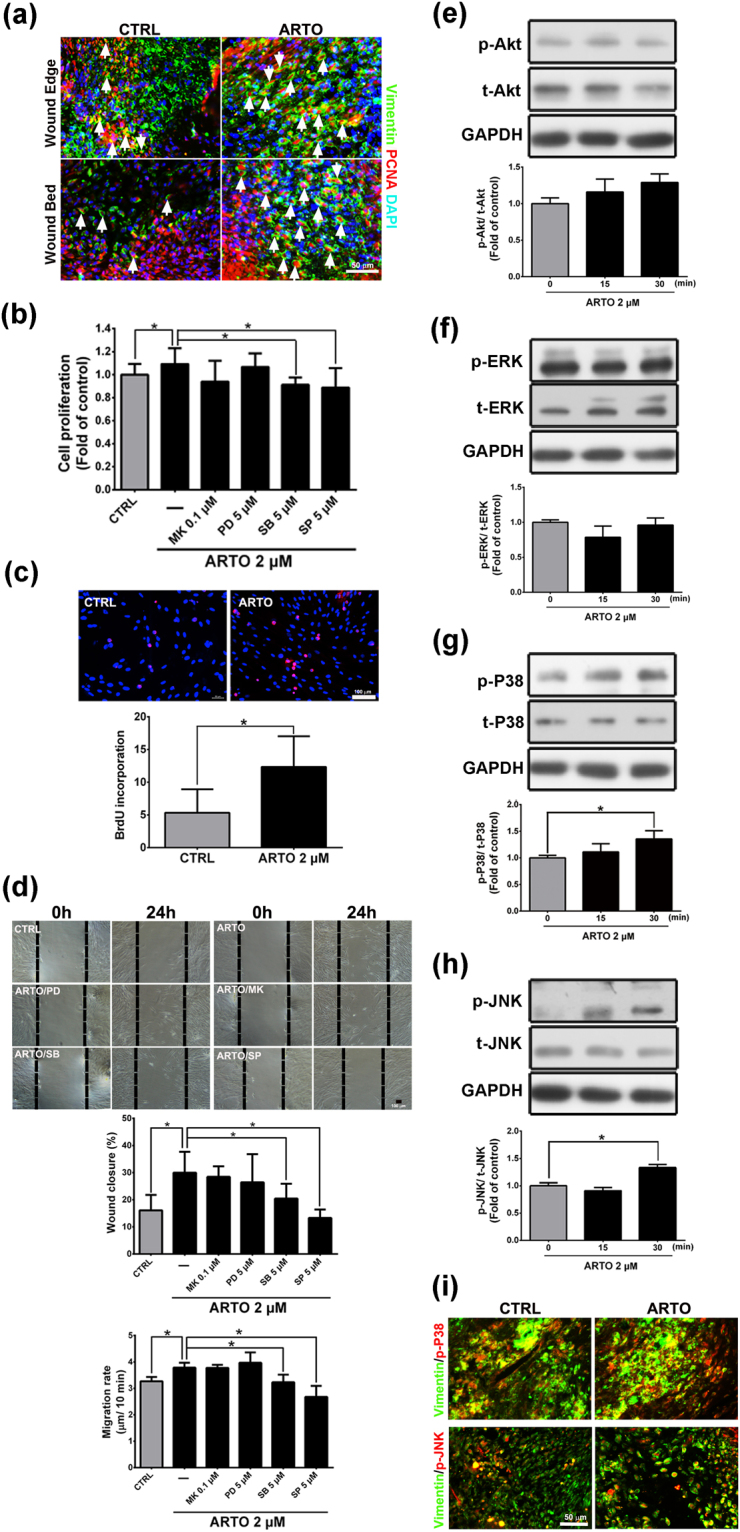
ARTO enhances human fibroblast proliferation and migration through the P38 or JNK signaling pathway. (**a**) Immunohistochemistry was performed to identify vimentin and PCNA (arrows) in wounds on day 15. The cells were pre-treated with MK2006 (MK), PD98059 (PD), SB203580 (SB), or SP600125 (SP) for 1 h and then incubated with ARTO for 24 h. Crystal violet staining (**b**) and a BrdU incorporation assays (**c**) were used to measure GM05386 cell number and proliferation. (**d**) Cell migration was examined via wound healing assays, in which GM05386 cells were wounded by scratch injury (black lines). The wound closure and migration rates were determined. The levels of Akt (**e**), ERK (**f**), P38 (**g**), and JNK (**h**) were determined by western blot analysis. (**i**) There was co-localization between vimentin and phosphorylated P38 or JNK in the wounds on day 15. The data are shown as the means ± S.D. N = 3–6 group and *P < 0.05.

An important process in the proliferative phase involves fibroblast proliferation and migration. In this study, immunohistochemistry showed that a higher proportion of co-localization of vimentin (a fibroblast marker) and proliferating cell nuclear antigen (PCNA) was present in the wound bed and wound edge in the ARTO-treated group than the control group on day 7 (Fig. 4a). To characterize the effects of ARTO on fibroblasts, we first observed the proliferation and migration of GM05386 fibroblasts and found that the total cell number (Fig. 4b), number of BrdU-positive cells (Fig. 4c), wound closure percentage, and migration rate (Fig. 4d) were significantly higher in the 2 μM ARTO group than the control group. These results indicated that ARTO enhances human fibroblast proliferation and migration.

To investigate the molecular mechanisms of the ARTO-induced increase in fibroblast proliferation, we observed the activation of Akt and MAPKs in GM05386 fibroblasts. Western blot analysis showed that the phosphorylation of P38 and JNK, but not Akt and ERK, significantly increased after the addition of ARTO (Fig. 4e–h). In addition, there was increased co-localization of phosphorylated P38 or JNK and vimentin in the ARTO-treated wound skin than the control wound skin (Fig. 4i). We next used crystal violet staining to determine whether the increase in the phosphorylation of these proteins contributed to the increase in GM05386 fibroblast proliferation. For this purpose, the cells were first treated with an inhibitor of Akt (MK2006, MK), ERK (PD98059, PD), P38 (SB203580, SB), or JNK (SP600125, SP) 1 h before ARTO treatment. The results showed that fibroblast proliferation was significantly higher in the 2 μM ARTO group without inhibitors (Fig. 4b). However, when SB203580 or SP600125 was present, the ARTO-induced increase in proliferation was suppressed in GM05386 fibroblasts. In addition, similar data were observed in scratch wound assays in GM05386 fibroblasts (Fig. 4d). Together, these data suggested that ARTO may enhance GM05386 fibroblast proliferation and migration through the P38 or JNK signaling pathways.

### ARTO enhances human keratinocyte proliferation and migration through the ERK and P38 signaling pathways

**Figure 5:**
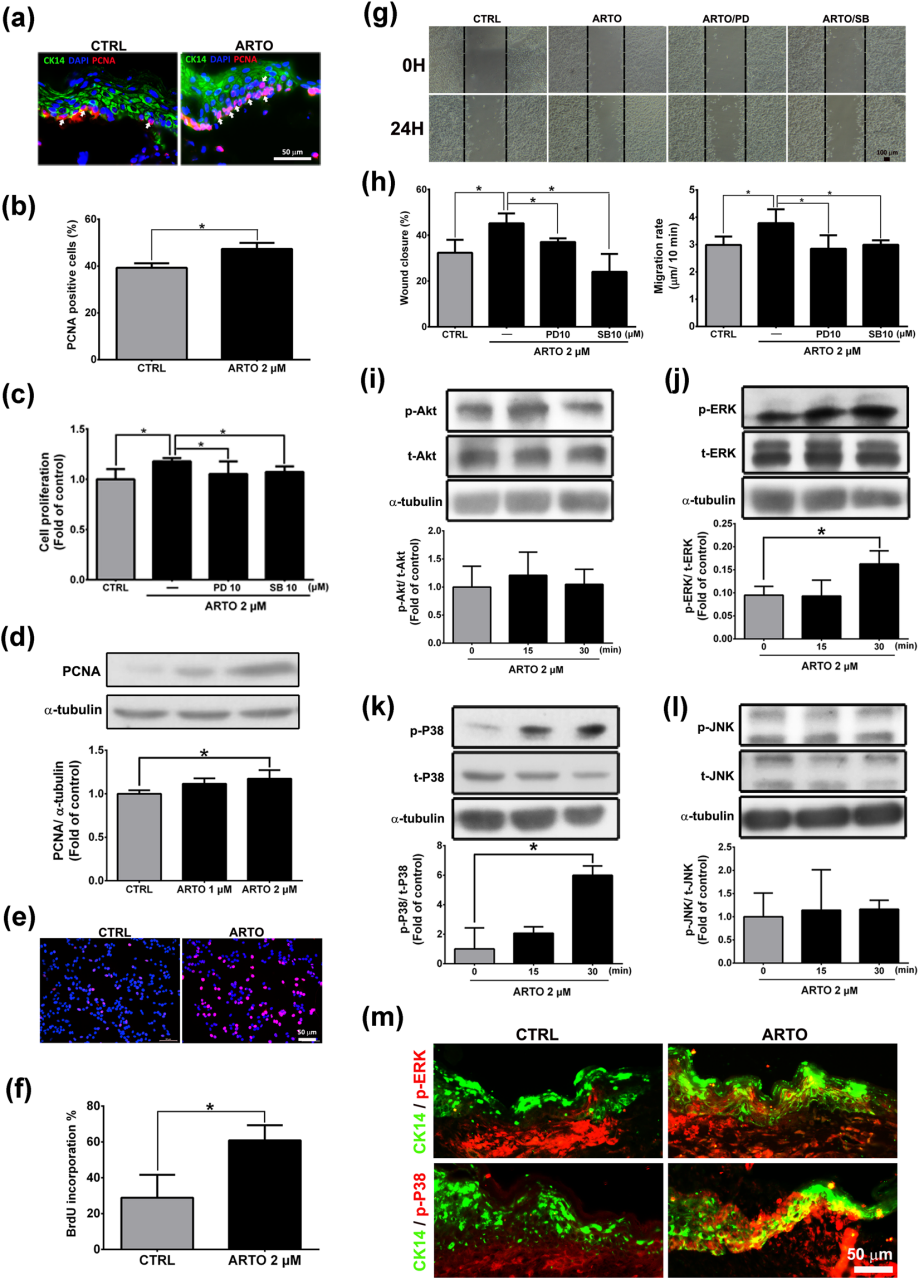
ARTO enhances human keratinocyte proliferation and migration through the ERK or P38 signaling pathway. (**a**) Wounded sections were stained with CK14 and PCNA (arrows). (**b**) Quantitative analysis of PCNA-positive cells. The cells were pre-treated with PD98059 (PD) or SB203580 (SB) for 1 h and then incubated with ARTO for 24 h. Crystal violet staining (**c**), western blot analysis (**d**), and BrdU incorporation assays (**e**) were used to measure keratinocyte proliferation. (**f**) Quantitative analysis of BrdU-positive cells. (**g**,**h**) The wound closure and migration rates were examined via wound healing assays, in which keratinocytes were wounded by scratch injury (black lines). The levels of Akt (**i**), ERK (**j**), P38 (**k**), and JNK (**l**) were determined by western blot analysis. (**m**) There was co-localization between CK14 and phosphorylated ERK or P38 in the wounds on day 7. The data are shown as the means ± S.D. N = 3–6 wounds/group and *P < 0.05.

Another important process in the proliferative phase is re-epithelialization, which involves keratinocyte proliferation and migration. Here, immunohistochemistry showed that a higher proportion of co-localization of CK 14 (a keratinocytes marker) and proliferating cell nuclear antigen (PCNA) was present in the basal layer of the epidermis in the ARTO-treated group than the control group on day 7 (Fig. 5a,b). To study the proliferation and migration effect of ARTO on human keratinocytes, crystal violet staining (Fig. 5c), western blot analysis (Fig. 5d), BrdU incorporation assays (Fig. 5e,f), and scratch wound assays (Fig. 5g,h) were performed. The results showed that the proliferation and migration of human keratinocytes were significantly higher in the 2 μM ARTO group than the control group. These data suggested that ARTO enhances keratinocyte proliferation and migration.

To investigate the molecular mechanisms underlying the ARTO-induced increase in the proliferation and migration of keratinocytes, we observed the activation of Akt and MAPKs in human keratinocytes. Western blot analysis showed that the phosphorylation of ERK and P38 significantly increased after the addition of ARTO (Fig. 5i–l). In addition, there was greater co-localization between phosphorylated ERK or P38 and CK14 expression in the ARTO-treated wound skin than the control skin (Fig. 5m). To further determine whether the increase in the phosphorylation of ERK and P38 contributed to the increase in human keratinocyte proliferation and migration, crystal violet staining and scratch wound assays were performed. For these purposes, the cells were pre-treated with PD98059 or SB203580 1 h before ARTO treatment, and the results showed that cell proliferation (Fig. 5c) and migration (Fig. 5g,h) were significantly higher in the 2 μM ARTO group without inhibitors. The increased proliferation and migration were inhibited by PD98059 and SB203580 pretreatment in human keratinocytes (Fig. 5c,g,h). These results suggested that ARTO enhances keratinocyte proliferation and migration through the ERK or P38 signaling pathway.

### ARTO enhances angiogenesis through the Akt and P38 signaling pathways

**Figure 6:**
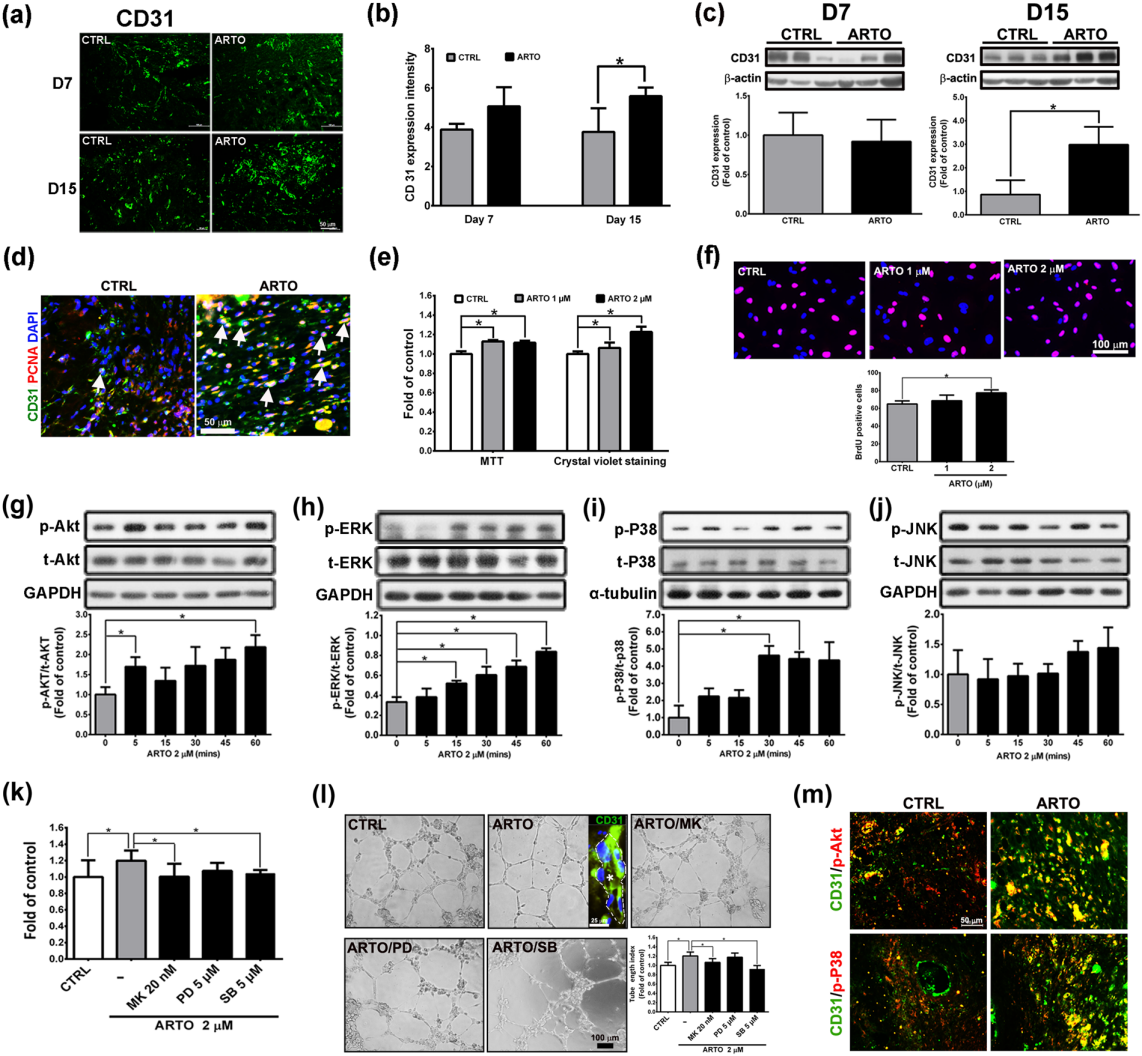
ARTO enhances angiogenesis through the Akt or P38 signaling pathway. (**a**,**b**) Wounded sections were stained with CD31, and a quantitative analysis of CD31 level was conducted. (**c**) Western blot analysis of the skin showed that CD31 level was increased in the ARTO-treated wounds on day 15 after wounding. (**d**) Immunohistochemistry was performed to identify CD31 and PCNA (arrows) in wounds on day 15. (**e**,**f**) MTT, crystal violet staining, and BrdU incorporation assays were used to measure cell viability and proliferation. The levels of Akt (**g**), ERK (**h**), P38 (**i**), and JNK (**j**) were determined by western blot analysis. HUVECs were pre-treated with Akt or MAPK inhibitors for 1 h and then incubated with ARTO for 24 h. (**k**) Crystal violet staining was performed. (**l**) Tube formation and tube length were examined by a Matrigel assay. Confocal image of a tube stained for CD31 (green) and cell nuclei (DAPI, blue). The asterisk indicates the lumen of the tube. (**m**) There was co-localization between CD31 and phosphorylated Akt or P38 in the wounds on day 15. The data are shown as the means ± S.D. N = 3–6 wounds/group and *P < 0.05.

During the wound healing process, angiogenesis is necessary to provide nutrients and oxygen to the wound area and new tissue^19^. In the present study, immunohistochemistry (Fig. 6a,b) and western blotting (Fig. 6c) showed that CD31 (an endothelial cell marker) level was significantly higher in the ARTO-treated group than the control group on day 15. Here, immunohistochemistry showed that a higher proportion of proliferating endothelial cells was present in the wound bed in the ARTO-treated group than the control group on day 15 (Fig. 6d). In an *in vitro* study, human umbilical vein endothelial cells (HUVECs) were subjected to MTT assays, crystal violet staining, and BrdU-based immunocytochemistry to determine the viability and proliferative effects of ARTO on endothelial cells. The results showed that 2 μM ARTO had no cytotoxicity toward HUVECs, and HUVEC proliferation was significantly higher in the 2 μM ARTO group than the control group (Fig. 6e,f). These results suggested that ARTO enhances endothelial cell proliferation and angiogenesis.

To investigate the molecular mechanisms of the ARTO-induced increase in endothelial cell proliferation and angiogenesis, we observed the activation of Akt and MAPKs in ARTO-treated HUVECs. Western blot analysis showed that the phosphorylation of Akt, ERK, and P38, but not JNK, significantly increased after ARTO treatment (Fig. 6g–j). To further determine whether the increase in the phosphorylation of these proteins contributed to the increase in HUVEC proliferation and angiogenesis, crystal violet staining and Matrigel tube formation assays were performed, and the results showed that cell proliferation and tube length were significantly higher in the 2 μM ARTO group without inhibitors (Fig. 6k,l). And the vascular-like tube with a visible lumen was seen in the matrigel (Fig. 6l). In addition, there was higher co-localization of phosphorylated Akt or P38 and CD31 expression in the ARTO-treated wound skin than the control skin (Fig. 6m). However, when MK2006 or SB203580 was present, the ARTO-induced increase in proliferation and tube length was suppressed (Fig. 6k,l). Together, these data suggested that ARTO enhances endothelial cell proliferation and tube formation through the Akt or P38 signaling pathway.

## Discussion

In this investigation, ARTO was found to enhance skin wound healing in C57BL/6 mice *in vivo* by inducing the inflammatory phase in the wound earlier and promoting collagen deposition, collagen maturation, myofibroblast differentiation, TGF-β production, keratinocyte proliferation, wound contraction, and angiogenesis in the wound tissue. Additionally, *in vitro* studies demonstrated that ARTO enhances TGF-β production, collagen level, and myofibroblast differentiation in fibroblasts and increases the proliferation and migration of human fibroblasts through the P38 or JNK pathway. ARTO increases the proliferation and migration of human keratinocytes through the ERK or P38 pathway. Moreover, ARTO enhances endothelial cell proliferation and tube formation through the Akt or P38 pathway.

The inflammatory phase is an essential event in wound healing for homeostasis maintenance and recruitment of the innate immune system to attack invading pathogens and remove dead tissues. Previous studies have shown that the number of macrophages that migrate to and infiltrate a wound peaks on day 3 after wounding and persists until day 7, and that the number of neutrophils in the wound peaks 12 h after wounding and then declines on day 3^20–23^. In the present study, immunohistochemical analysis of neutrophils and macrophages revealed that a large number of inflammatory cells were already present at the wound site in the ARTO-treated group on day 1 and that the number declined dramatically on day 3, whereas a large number of inflammatory cells appeared on day 3 in the control group, thus indicating an early peak of inflammation on day 1 or even earlier in the ARTO-treated group. These data suggested that ARTO induces the inflammatory phase earlier and thus accelerates the wound healing process. Numerous cytokines involves in wound healing process. MCP-1, also known as chemokine (C-C motif) ligand 2 (CCL2), attracts monocytes, memory T cells, and dendritic cells during inflammation^24,25^. MIP-2, also known as chemokine (C-X-C motif) ligand 2 (CXCL2), attracts granulocytes (including neutrophils) and hematopoietic stem cells^26^. Moreover, C5/C5a, which is part of the complement system, is involved in attracting immune cells, and a previous study has shown that decreased level of C5/C5a enhances wound healing^27^ and that MCP-1 and MIP-2 are highly expressed on day 1 after wounding^28^. In the present study, we also noted lower C5/C5a, MCP-1, and MIP-2 level in the ARTO-treated group than the control group on day 1 via a cytokine membrane array assay. Because the level of the chemoattractant cytokines was lower on day 1 after wounding, fewer neutrophils and macrophages infiltrated ARTO-treated wounds than control wounds on day 3 after wounding. A previous study has similarly shown that ARTO enhances the migration of neutrophils^29^, which may be one of the mechanisms of the accelerated infiltration and elimination of neutrophils and macrophages that accelerates the wound healing process.

Inflammation is a crucial event in the wound healing process. Successful wound repair requires inflammation resolution after wound injury, whereas excessive inflammation results in chronic wounds and scar formation^30^. In the present study, we noted lower C5/C5a, MCP-1, MIP-1α, IL-16, IL-1β, and MIP-2 level in the ARTO-treated group than the control group on day 1 or day 3 via a cytokine membrane array assay. IL16 and IL-1β are chemoattractant for white blood cells^31,32^. A previous study has similarly shown that ARTO exhibits anti-inflammatory effects by decreasing IL-1β expression^9^ and inhibiting lipopolysaccharide-induced nitric oxide (NO) production^10^. These data suggest that ARTO exerts anti-inflammatory effects.

ECM deposition, maturation, and reorganization are important events during the wound healing process. Matrix metalloproteinases (MMPs), a group of enzymes responsible for ECM degradation, play an important role in regulating ECM turnover and homeostasis. The tissue inhibitors of metalloproteinases (TIMPs) are specific inhibitors of MMPs that bind to the active MMP and then inhibit the ECM degradation process. A previous study has shown that excess TIMP-1 expression may contribute to impaired epithelial cell migration, an important event in re-epithelialization, and subsequently hamper wound healing^33,34^. In the present study, we found lower TIMP-1 level in the ARTO-treated group than the control group on days 1 and 3 via a cytokine membrane array assay. Additionally, we found that ARTO enhances re-epithelialization. On the basis of these results, we propose that ARTO enhances re-epithelialization and wound healing by decreasing TIMP-1 level during wound remodeling from day 1 to day 3 after wounding.

Wound healing is a dynamic and complex process including three main overlapping phases: the inflammatory phase, the proliferative phase, and the remodeling phase. In the present study, mice were used as an animal model to evaluate the therapeutic effects of artocarpin on wound healing. However, the regulatory mechanisms of the wound healing process between human and mice are different. Re-epithelialization and granulation tissue formation play key roles in the wound healing process in humans, whereas contraction is the most important regulator during the wound healing process in loose-skinned mice^35,36^. To avoid and restrict automatic wound contraction in mice, splinting silicone was used in the present study^36,37^. Compared with another study^38^, wounds with splinting silicone have been found to be incompletely closed on day 15 after wounding, similarly to the present results. In addition, the amount and distribution of collagen bundles also have a major influence on the shape and degree of wound closure^39^. Fewer mature, much thinner, and non-uniform arrangements of collagen fiber were observed in the control-treated skin than ARTO-treated wound skin (Fig. 3b–d), and a higher wound closure percentage was also seen in the ARTO-treated group than in the control group on day 15 after wounding (Fig. 1b,c). Together, these results indicated that delayed wound closure in the control group may have been due to the synthesis, maturation, and arrangement of collagen fibers.

A previous study has shown that ARTO exerts cytotoxicity on HaCaT keratinocytes at a high concentration (10 µM) in *in vitro* studies and at 0.1% in *in vivo* studies^9^. The 0.05% ARTO dose has been found to be the proper and safe dose for the topical formulation to be protective against UVB irradiation-induced injury^9^. In the present study, 1-2 µM ARTO was used in *in vitro* studies, and 0.08% ARTO was used in *in vivo* studies. At these ARTO doses, ARTO exerts no cytotoxic effects on human keratinocytes, increases the proliferation and migration of keratinocytes in *in vitro* studies, and enhances the re-epithelialization in *in vivo* studies.

Wound contraction and ECM reorganization are important events during the wound healing process. The expression and differentiation of myofibroblasts are essential events during wound contraction, and excessive and prolonged myofibroblast expression in the wound area results in scarring and fibrosis^40,41^. Previous studies have also shown that TGF-β stimulates fibroblast differentiation into myofibroblasts in wounds^42,43^. In the present study, ARTO significantly increased the myofibroblasts level in the remodeling phase on days 3 and 7 after wounding. The increasing effect was decreased on day 15 after wounding (Fig. 1g). Moreover, we found a higher level of TGF-β in the ARTO-treated wounds than in the control wounds on day 7 after wounding. In addition, similar data were observed in GM05386 fibroblasts (Fig. 3e). On the basis of these results, we propose that ARTO enhances wound contraction and tensile strength by increasing TGF-β level during wound remodeling from day 3 to day 7 after wounding. However, this effect of myofibroblasts level diminished by day 15 after wounding, thus indicating that ARTO has a potential therapeutic effect on wound contraction but has no influence on scarring or fibrosis.

**Figure 7:**
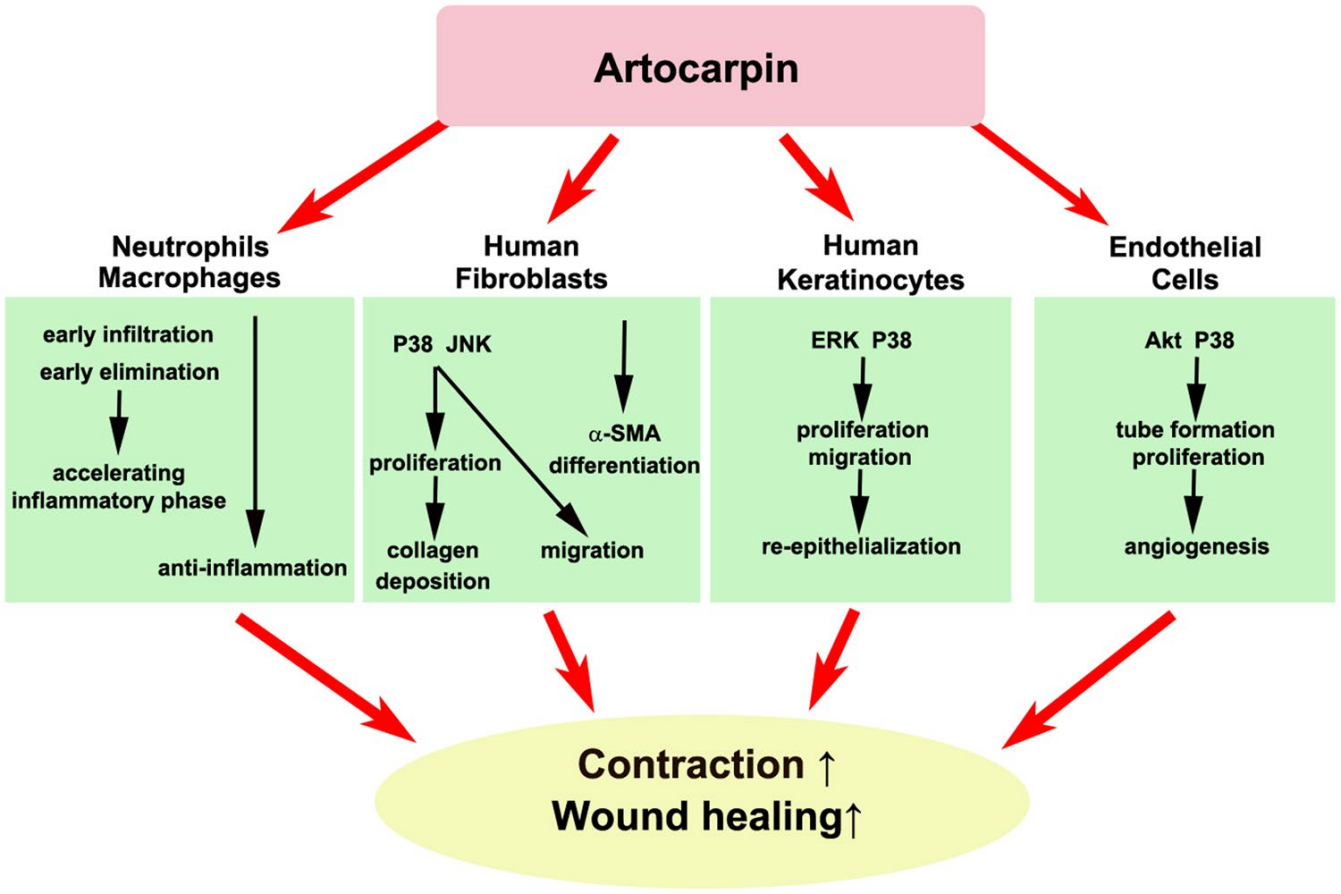
Graphical schematic of the regulatory mechanism of ARTO in enhancing skin wound healing.

In conclusion, in the present study, we observed that ARTO enhances skin wound healing through multiple mechanisms. In particular, ARTO results in early stimulation of the inflammatory phase and enhances the myofibroblast differentiation of fibroblasts; the proliferation and migration of fibroblasts, keratinocytes; the proliferation of endothelial cells and tube formation (Fig. 7). These effects then accelerate inflammatory progression and enhance collagen deposition, re-epithelialization, wound contraction, and angiogenesis. Therefore, we believe that ARTO may have therapeutic potential in the treatment of cutaneous wounds.

## Materials and Methods

### Reagents

Polyclonal rabbit IgG antibodies against human GAPDH, β-actin, phospho-/total-P38, phospho-/total-ERK1/2, phospho-/total-JNK, α-actin, collagen, phospho-/total-Akt, horseradish peroxidase (HRP)-conjugated goat anti-mouse IgG, and anti-rabbit IgG antibodies were purchased from GeneTex (Irvine, CA, USA). Polyclonal rabbit antibodies against human collagen were purchased from Proteintech (Chicago, IL, USA). Monoclonal rabbit antibodies against human PCNA were purchased from Santa Cruz Biotechnology (Santa Cruz, CA, USA). Monoclonal rabbit antibodies against human TGF-β and vimentin were purchased from Abcam (Cambridge, UK). Polyclonal rabbit IgG against human Iba-1 was purchased from Wako (Chuo-ku, Osaka, Japan). Polyclonal rabbit IgG against human MPO was purchased from MyBioSource (San Diego, CA, USA). MK2006, PD98058, SP600125, and SB203580 were purchased from Biomol (Plymouth Meeting, PA, USA). LPS and BrdU were purchased from Sigma-Aldrich (St. Louis, MO, USA). TRITC- and FITC-conjugated goat anti-mouse IgG antibodies were purchased from Jackson ImmunoResearch (West Grove, PA, USA).

### Extraction and purification of ARTO

ARTO was isolated from the dichloromethane fraction of *A*. *communis*. Briefly, the heartwood of *A*. *communis* was immersed in methanol. The methanol extract was then collected, filtered, concentrated, and lyophilized, and this was followed by suspension of the crude extract in water and its successive portioning with equivalent volumes of n-hexane, dichloromethane, ethyl acetate, and n-butanol. The organic solvent of each extract was removed by rotary vacuum evaporation. The purified fraction was subsequently recrystallized to obtain ARTO (Fig. 1a), and this purified ARTO was stored at −20 °C until use. The extracted ARTO was evaluated by high-performance liquid chromatography (HPLC) and determined to be 95% pure.

### Cell culture

Human keratinocytes (human oral keratinocyte 1; OK1) were kindly provided by Dr. W-W Chang. The GM05386 cells (human primary fibroblasts) were obtained from the Coriell Cell Repository (Camden, New Jersey). The HUVECs were obtained from the Bioresource Collection and Research Center (BCRC). Human keratinocytes were cultured in DMEM containing 10% FBS, 1 mM pyruvate, 2 mM L-glutamate, and a 1% antibiotic/antimycotic solution. The GM05386 cells were cultured in DMEM containing 10% FBS and a 1% antibiotic/antimycotic solution at 37 °C in a 5% CO_2_ atmosphere. The HUVECs were grown in Endothelial Cell Medium (Lonza, Walkersville, MD, USA) containing penicillin-streptomycin (1%) and endothelial cell growth supplement at 37 °C in a humidified atmosphere of 95% air and 5% CO_2_ and were used between passages 2 and 5.

### Cell viability and proliferation assays

The cells were treated with various concentrations of ARTO for 24 h. MTT (3-(4,5-dimethylthiazol-2-yl)-2,5-diphenyl tetrazolium bromide) assays were used to determined cell viability. Cell proliferation was determined using crystal violet staining.

### Wound healing assay

The cells were grown in 24-well plates. After cells were achieved confluence, wounds were inflicted by dragging a sterile pipette tip across the monolayer and then change fresh medium to remove the cell debris. After rinsing off released cells, the cells were cultured in the complete medium containing the ARTO and inhibitors. After the treatment, photos were taken in two regions along the wound. Time-lapse images were taken every 10 min after wounding over a 24 h period. The migration rate of cells was measured after collection of sequential time-lapse images. Analyses were performed on sequential phase contrast images with MetaMorph software. Only the cells near the wounded surface were chosen for migration rate determination. In each wound, 8–10 cells were tracked in each field, and all experiments were performed in triplicate. The conversion factor for measured pixels to microns (0.46 for 20 x objective) was determined. The percentage of the wound closure was determined by measuring the area of the wound at day 0, and measuring the area of migrated cell that filled that area at 24 h after wound. After 24 h, the wound closure area/original wound area ratio were calculated using Image-Pro Plus 4.5 software.

### BrdU incorporation assay

The cells were cultured on gelatin-coated coverslips. After pretreatment with ARTO and 10 mg/mL 5-bromo-2′-deoxyuridine (BrdU, Sigma-Aldrich) for 24 h, the treated cells were fixed and stained with an anti-BrdU antibody overnight at 4 °C, and then incubated with TRITC-conjugated goat anti-mouse IgG. The cells were counterstained with 1 µg/mL DAPI and observed under a fluorescence microscope.

### Mouse cytokine array

The wound tissue lysates were collected for cytokine secretion analysis. Cytokine level profiles were determined using a mouse cytokine membrane array (R&D Systems Inc., Minneapolis, MN, USA) according to the manufacturer’s instructions.

### Immunohistochemical staining

Wound tissues were fixed with 4% paraformaldehyde. The fixed tissues were subsequently blocked in 10% normal goat serum for 1 h, then incubated with the appropriate primary antibodies (all at 1:100) at 4 °C overnight. The tissues were then incubated with FITC-conjugated secondary antibody for 1 h at room temperature before being counterstained with DAPI and examined using a fluorescence microscope.

### Immunoblotting analysis

The proteins of wound skin (10-mm diameter) collected at the intervals of 3, 7, and 15 days after wounding were extracted using RIPA lysis buffer (Cell Signaling, Beverly, MA, USA) with added protease inhibitors (Sigma). The tissue proteins were normalized for total protein concentration using Bradford protein assays. The proteins (20 μg) of the wound tissue samples or cells were then subjected to sodium dodecyl sulfate polyacrylamide gel electrophoresis (SDS-PAGE) and transferred to polyvinylidene fluoride (PVDF) membranes. After the membranes were blocked with 5% BSA for 1 h, they were incubated with the appropriate primary antibodies (all at 1:1000) at 4 °C overnight, then incubated with HRP-conjugated secondary antibodies (all at 1:3000) for 1 h. Immunoreactivity was then detected with ECL. The intensities of the bands were quantified using Gel-Pro software. Rabbit anti-human GAPDH and β-actin antibodies were used as internal controls (both at 1:3000).

### Matrigel assay

Matrigel (Becton Dickinson) was thawed and mixed 1:1 with serum-free media and dispersed onto 96-well plates at 37 °C for at least 1 h. Then, cells were trypsinized, and 10^4^ HUVECs were added to the gel matrix and incubated for 8 h. Tubule formation was inspected under an inverted light microscope. A modification protocol for hollow vascular-like tube observation was performed. In brief, HUVEC cells were plated into 24-well plates containing coverslips. After cells attachment, the cells were overlaid with Matrigel. After gel formation, the gels were overlaid with culture media containing ARTO for 24 h. After 24 h of culture, the cells were fixed and stained for CD31 overnight and then incubated with FITC-conjugated goat anti-rabbit IgG. The hollow vascular-like tubes were observed by confocal microscope.

### Excisional wound model

Male C57BL/6 (8 wk) mice were purchased from the National Laboratory Animal Center (Taipei, Taiwan). All of the procedures were performed in accordance with the local institutional guidelines for animal care established by National Taiwan University and complied with the “Guide for the Care and Use of Laboratory Animals” NIH publication No. 86–23, revised in 2011. The protocol was also approved by the National Taiwan University College of Medicine and College of Public Health Institutional Animal Care and Use Committee (IACUC NO: 20150293). Mice were anesthetized by intraperitoneal injection of pentobarbital at 70 mg/kg body weight, and two 6-mm wounds were made in their dorsal skin. A 10-mm-diameter silicone ring was then placed and sutured around the perimeter of each wound to restrict contraction. The mice were randomized into the following two groups: a control group (vehicle control), which received 10 µl of DMSO, and an ARTO group. In total, 20 µM ARTO in 10 µl of DMSO was administered daily to each mouse by topical application. The wound area was determined by taking daily digital pictures of the wounds. The mice were sacrificed after 1, 3, 7, or 15 days via an overdose of pentobarbital, and the wound skin samples were collected and fixed in 4% paraformaldehyde, embedded, and sectioned for morphometric analysis and immunohistochemistry. The wound skin samples were serially cut into 5-µm sections, and every tenth section was stained with H&E (Sigma-Aldrich), Mallory’s trichrome (Sigma-Aldrich), and picrosirius red (Sigma-Aldrich).

### Transmission electron microscopy (TEM)

The specimens were fixed in 2% glutaraldehyde and 2% paraformaldehyde and then treated with 1% osmium tetroxide for 1 h. The wound skin samples were subsequently embedded in Epon and processed for TEM (HITACHI H-700).

### Statistical analyses

All of the values are presented as the means ± SD. Statistical comparisons were performed using a two-tailed Student’s t-test or one-way analysis of variance (ANOVA). Nonparametric statistical tests were used in this study. Comparisons between the two groups were performed with the Mann-Whitney test. *P-*values < 0.05 were considered significant.
